# Learning to optimize perceptual decisions through suppressive interactions in the human brain

**DOI:** 10.1038/s41467-019-08313-y

**Published:** 2019-01-28

**Authors:** Polytimi Frangou, Uzay E. Emir, Vasilis M. Karlaftis, Caroline Nettekoven, Emily L. Hinson, Stephanie Larcombe, Holly Bridge, Charlotte J. Stagg, Zoe Kourtzi

**Affiliations:** 1 Department of Psychology, University of Cambridge, Cambridge, CB2 3EB UK; 2 Purdue University School of Health Sciences, 550 Stadium Mall Drive, West Lafayette, IN 47907 USA; 3 Wellcome Centre for Integrative Neuroimaging, FMRIB, Nuffield Department of Clinical Neurosciences, University of Oxford, Oxford, OX3 9DU UK; 4 Oxford Centre for Human Brain Activity, Wellcome Centre for Integrative Neuroimaging, Department of Psychiatry, University of Oxford, Oxford, OX3 7JX UK

## Abstract

Translating noisy sensory signals to perceptual decisions is critical for successful interactions in complex environments. Learning is known to improve perceptual judgments by filtering external noise and task-irrelevant information. Yet, little is known about the brain mechanisms that mediate learning-dependent suppression. Here, we employ ultra-high field magnetic resonance spectroscopy of GABA to test whether suppressive processing in decision-related and visual areas facilitates perceptual judgments during training. We demonstrate that parietal GABA relates to suppression of task-irrelevant information, while learning-dependent changes in visual GABA relate to enhanced performance in target detection and feature discrimination tasks. Combining GABA measurements with functional brain connectivity demonstrates that training on a target detection task involves local connectivity and disinhibition of visual cortex, while training on a feature discrimination task involves inter-cortical interactions that relate to suppressive visual processing. Our findings provide evidence that learning optimizes perceptual decisions through suppressive interactions in decision-related networks.

## Abstract (web summary)

Learning improves perceptual decisions by enhancing the brain's ability to filter noise and irrelevant information. Here, the authors show that GABAergic inhibition in decision-making circuits supports our ability to optimize perceptual judgments through learning and experience.

## Introduction

Making successful decisions entails extracting meaningful information from multiple sources in the environment that are inherently noisy and ambiguous. Experience and training have been shown to play a key role in optimizing perceptual decisions^1–3^ by filtering external noise (e.g., when detecting targets in cluttered scenes) and retuning task-relevant feature templates (e.g., when discriminating highly similar objects)^4–6^. Previous functional magnetic resonance imaging (fMRI) studies have demonstrated learning-dependent changes in functional brain activity due to training on perceptual tasks that involve detecting targets in clutter or discriminating fine feature differences (for reviews^7,8^). However, fMRI does not allow us to distinguish excitatory from inhibitory mechanisms of experience-dependent plasticity, as BOLD reflects aggregate activity from both excitatory and inhibitory signals across large neural populations^9^. Thus, the inhibitory brain plasticity mechanisms that support our ability to improve our perceptual decisions by learning to suppress noisy and task-irrelevant information through training remain largely unknown.

To investigate inhibitory mechanisms of learning-dependent plasticity, we employed magnetic resonance spectroscopy (MRS) that has only recently made it possible to measure γ-aminobutyric acid (GABA), the primary inhibitory neurotransmitter in the brain. Previous animal studies have linked decreased GABAergic activity to learning and synaptic plasticity in primary motor cortex^10,11^. In accordance with these findings, human MRS studies have shown that GABA levels in the primary motor cortex decrease following interventions that facilitate cortical reorganisation^12^ and motor training^13,14^. In the visual cortex, human MRS studies have shown that GABA levels relate positively to performance in perceptual tasks^15–17^. Further, decrease in visual GABA has been shown to relate to homeostatic plasticity^18^. Here, we took advantage of the high spectral resolution afforded by ultra-high field (7T) MRS to reliably resolve GABA^19,20^ and take fast and reliable repeated measurements of functional GABA during training. This allowed us to test changes in GABA during training (i.e., while the participants were trained on a task), extending beyond standard correlational approaches that relate single measurements of GABA at baseline (i.e., when participants are at rest) to behavior. Further, we tested whether changes in GABAergic inhibition during task-specific training relate to improvement in perceptual decisions.

To probe the brain mechanisms that support learning by suppressing noisy and irrelevant signals, we employed two learning tasks that have been shown to rely either on noise filtering or feature template retuning: (1) a signal-in-noise task that involves extracting a target masked by noise, (2) a feature differences task that involves judging fine differences^21^. Recent computational investigations^22,23^ and animal studies propose dissociable roles for inhibition in learning to interpret noisy sensory signals vs. tuning fine feature processing. Based on this work, we hypothesized that distinct GABAergic inhibition mechanisms are involved in task-dependent learning and plasticity. Specifically, we reasoned that decreased GABAergic inhibition during training would relate to improved ability to detect targets in clutter, as changes in GABAergic inhibition have been linked to neural gain, (i.e., changes in information transmission between neurons^24^ or the slope of the neural input-output relationship^25^). In contrast, we reasoned that increased GABAergic inhibition would relate to improved performance in fine feature discrimination, as increased GABAergic inhibition has been linked to enhanced orientation selectivity in visual cortex^16,26–28^.

Further, previous neuroimaging and neurophysiology studies have implicated distinct functional roles for the visual and posterior parietal cortex (PPC) in sensory processing vs. perceptual decision making, respectively^29,30^. To test the role of inhibitory processing in learning for both visual and parietal cortex, we implemented an imaging protocol that measured GABA in two voxels (one in occipito-temporal (OCT), one in PPC) in alternating order and allowed us to track changes in GABA in both areas during training. Interestingly, previous studies have proposed that perceptual learning is implemented by top-down influences from decision-related areas that re-weight processing in sensory areas^30,31^. To test whether learning involves local processing within visual cortex or suppressive interactions between decision-related and sensory areas, we combined GABA measurements in occipito-temporal and posterior parietal cortex with functional brain connectivity, as measured by resting-state fMRI. In particular, we tested the hypothesis that learning is implemented by local inhibitory processing in visual cortex that is gated by functional interactions between sensory and decision-related areas. Specifically, we tested whether learning-dependent changes in visual cortex GABA relate to functional connectivity between visual and posterior parietal cortex.

Our results reveal distinct GABAergic inhibition mechanisms in a cortical network that is known to be involved in perceptual decisions. In particular, increased parietal GABA with training suggests suppression of task-irrelevant information. In contrast, changes in occipito-temporal GABA with training relate to enhanced target detection and discriminability, suggesting learning-dependent changes in the processing of task-relevant features. Further, analysis of functional brain connectivity at rest reveals interactions within this network that relate to GABA changes and behavioral improvement during training. Learning to detect targets from clutter is implemented by local connectivity and disinhibition of the visual cortex. In contrast, learning feature differences is implemented by interactions between parietal and visual areas that relate to increased GABAergic inhibition in visual cortex. Our results provide evidence that learning improves perceptual decisions through suppressive interactions within decision-related circuits in the human brain.

## Results

### Training improves behavioral performance

**Fig. 1:**
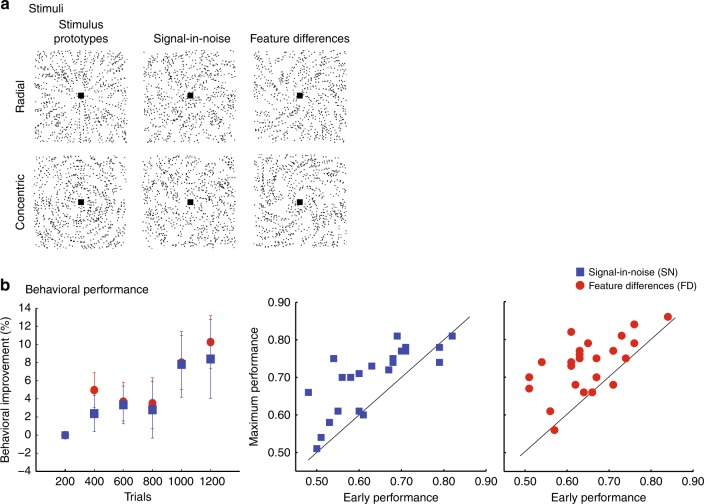
Tasks, stimuli, and behavioral results. **a** Stimuli: Example stimuli comprising radial and concentric Glass patterns (stimuli are presented with inverted contrast for illustration purposes). Stimuli are shown for the signal-in-noise task (SN task, 25% signal, spiral angle 0° for radial and 90° for concentric) and the feature differences task (FD task, 100% signal, spiral angle 38° for radial and 52° for concentric). Prototype stimuli (100% signal, spiral angle 0° for radial and 90° for concentric) are shown for illustration purposes only. **b** Behavioral performance across participants: We calculated improvement in task performance during training as the difference in mean performance (i.e., mean accuracy) during each training block (200 trials) from the first training block (200 trials), divided by performance in the first training block. Further, we compared individual participant accuracy early in training (first 200 trials) to the maximum accuracy achieved per participant during training (200 trials). Participants did not differ significantly in their performance early in training (*t*(45) = 0.56, *p* = 0.57) between the two tasks. Note that most participants (85%) achieved maximum performance during the last two MRS measurements. Error bars indicate standard error of the mean across participants

We tested two groups of participants on (a) a signal-in-noise (SN) task that involves extracting shapes (radial vs. concentric Glass patterns) masked by noise or (b) a feature differences (FD) task that involves judging fine differences induced by morphing between two stimulus classes (Fig. 1a). Participants were asked to judge the identity of the stimulus presented per trial (i.e., radial vs. concentric). Our results showed that participants improved in their judgments within a single training session during scanning (Fig. 1b), consistent with previous reports showing fast behavioral improvement early in the training (for a review, see ref. ^32^). A linear mixed effects (LME) model with Task and Training Block (6 blocks, 200 trials per block) as fixed effects showed significantly improved performance during training across tasks (main effect of Block: *F*(1, 249) = 9.35, *p* = 0.002). No significant interaction between Task × Block (*F*(1, 249) = 0.10, *p* = 0.75) was observed, suggesting similar improvement across tasks (Fig. 1b).

To quantify behavioral improvement due to training in individual participants, we compared performance at the beginning of training (i.e., first training block) to the maximum performance achieved (i.e., when participants achieved highest accuracy) by each participant during training (Fig. 1b) (see Supplementary Methods). We chose this measure to capture individual variability across participants that may be more pronounced in our data, as participants were trained only for a single training session in contrast to our previous studies that have shown that performance saturates after multiple training sessions on similar perceptual tasks^33,34^. A repeated measures two-way analysis of variance (Task × Block) showed significantly improved performance during training across tasks (main effect of block: *F*(1, 45) = 59.88, *p* < 0.0001) but no significant interaction between Task × Block (*F*(1, 45) = 1.07, *p* = 0.31).

### Learning-dependent changes in GABA

**Fig. 2:**
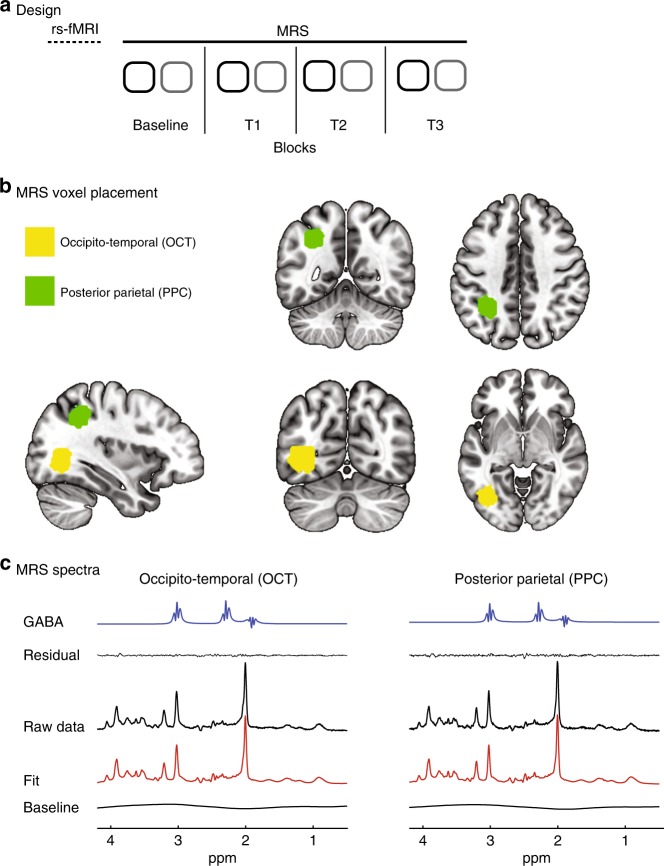
Task design, and MR spectroscopy (MRS) voxels. **a** Design: Each participant took part in a single session during which we acquired resting-state functional MRI (rs-fMRI) and MRS data. We collected MRS GABA during one block before (Baseline) and three blocks during training (T1, T2, T3). Each block comprised two MRS acquisitions: one from occipito-temporal (OCT voxel—black squares) and one from posterior parietal (PPC voxel—gray squares) cortex. The order of the voxels within each block was counterbalanced across participants. During each block, participants were presented with stimuli for 400 trials (200 trials per MRS voxel acquisition). **b** MRS voxel placement: We positioned the MRS voxels using anatomical landmarks (superior temporal gyrus and middle occipital gyrus for OCT and intraparietal sulcus for PPC) on the acquired T1 scan to ensure that voxel placement was consistent across participants. The mean GM tissue fraction was 44 ± 8% for OCT and 46 ± 7% for PPC and GM tissue content did not differ significantly between the two MRS Voxels (*t*(81) = 1.17, *p* = 0.24). The average distance of individual MRS voxels from the mean Montreal Neurological Institute (MNI) coordinates (OCT: *x* = −38.6 ± 4.4 mm, *y* = −67.8 ± 4.8 mm, *z* = 1.8 ± 4.4 mm; PPC: *x* = −31.6 ± 4.0 mm, *y* = −50.5 ± 6.5 mm, *z* = 40.7 ± 4.7 mm) across participants was small (i.e., lower than the MRS spatial resolution; 6.9 ± 3.6 mm for OCT and 7.9 ± 4.0 mm for PPC and did not differ between the two tasks (*t*(34) = 0.02, *p* = 0.99 for OCT; *t*(38) = 0.65, *p* = 0.52 for PPC). We computed the overlap across participant MRS voxels for OCT (yellow) and PPC (green) separately. We illustrate a group MRS mask (sagittal, coronal, axial view) that covers a cortical area that is common in at least 50% of the participants’ MRS voxels. **c** MRS spectra: Example spectra from the OCT and PPC voxel for one participant (see Supplementary Figure 1 for an average MRS spectrum across participants and Supplementary Figure 2 for individual participant MRS spectra). We show the GABA fit using LC model (GABA peaks at 1.89 ppm, 2.29 ppm and 3.01 ppm), the residuals, the spectrum comprising all metabolites, the LC Model fit, and the baseline

To test whether GABAergic inhibition in the visual or parietal cortex changes with training, we measured GABA (Fig. 2a) before (baseline block) and during training. During the baseline block, participants were presented with random dot stimuli, while during the training blocks participants performed either the SN or the FD task on Glass pattern stimuli. We tested two MRS voxels—one centered on the occipito-temporal cortex (OCT voxel) and the other on the PPC (PPC voxel) (Fig. 2b)—following previous studies showing that these areas are involved in learning using the same tasks and stimuli as in our study^33,34^. Each block comprised one MRS acquisition per voxel and the order of the voxels within each block was counterbalanced across participants.

**Fig. 3:**
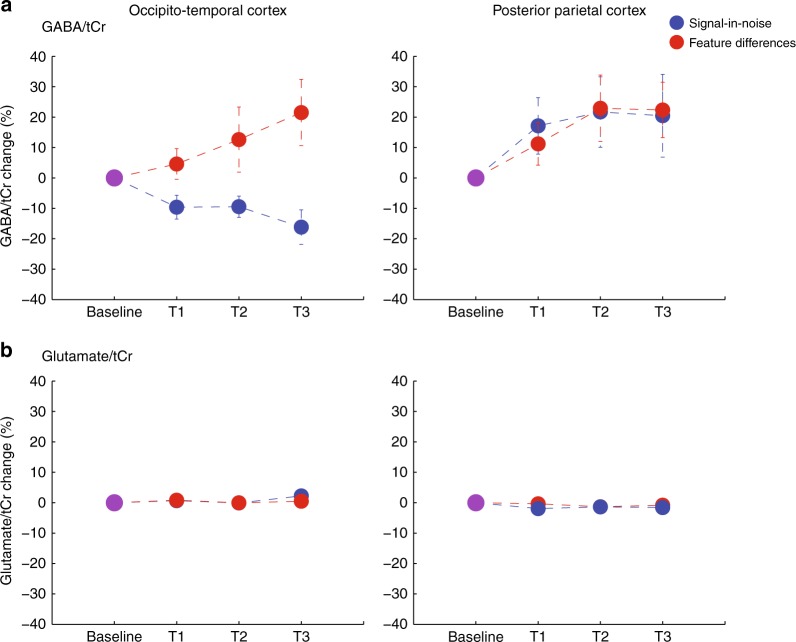
MR spectroscopy (MRS) measurements of γ-aminobutyric acid (GABA) and glutamate during training. **a** MRS-measured GABA over time is shown from two voxels (occipito-temporal, posterior parietal cortex) per task (signal-in-noise, feature differences). For each MRS voxel, we calculated % GABA change: we normalized GABA/tCr per training block (T1, T2, T3) to GABA/tCr recorded during the baseline block; that is, we computed GABA/tCr change subtracting GABA/tCr measurements in each of the three training blocks from the baseline block and then divided by GABA/tCr in the baseline block. We observed maximum (across blocks) 20% GABA change (mean across participants). This is consistent with previous studies measuring GABA at 3T that have reported changes in GABA between 10 and 15% within a single session (duration of 20–30 min) of stimulation^75^ or training^13^. Ultra-high field imaging (i.e., 7T) has been shown to have increased sensitivity for MR spectroscopy measurements^19,20^ and may result in enhanced and more accurate detection of learning-dependent changes in GABA. **b** MRS-measured glutamate over time is shown from two voxels (occipito-temporal, posterior parietal cortex) per task (signal-in-noise, feature differences). For each MRS voxel, we calculated % glutamate change: we normalized glutamate/tCr per training block (T1, T2, T3) to glutamate/tCr recorded during the baseline block; that is, we computed glutamate/tCr change subtracting glutamate/tCr measurements in each of the three training blocks from the baseline block and then divided by glutamate/tCr in the baseline block. Error bars indicate standard error of the mean across participants. Error bars are not visible for small changes in metabolite concentrations

Figure 3 shows that OCT GABA/tCr changed in the two tasks during training in opposite directions. In contrast, PPC GABA/tCr changed in the same direction (i.e., increased during training) for both tasks. These effects were supported by an LME analysis (Task, Voxel, and MRS Blocks as fixed effects) that modeled GABA before (baseline block) and during (three training blocks) training in both OCT and PPC. This analysis showed that learning-dependent changes in GABA levels differed between tasks and regions (Task × Voxel × Block: *F*(1, 264) = 6.24, *p* = 0.01). In particular, we found that changes in OCT GABA (OCT GABA) levels during training differed between tasks (LME model for OCT GABA with Task and MRS Block as fixed effects; Task × Block: *F*(1, 119) = 10.77, *p* = 0.001) (Fig. 3a). In contrast, GABA changes in the PPC (PPC GABA) during training did not differ significantly between tasks (LME model for PPC GABA with Task and MRS Block as fixed effects; Task × Block: *F*(1, 145) = 0.18, *p* = 0.68) (Fig. 3a). That is, PPC GABA increased during training independent of Task (LME model for PPC GABA with MRS Block as fixed effect; main effect of Block: *F*(1, 147) = 6.27, *t* = 2.50, *p* = 0.01).

Specifically, for the FD task, GABA changes during training did not differ significantly between OCT and PPC (LME model for FD task GABA with Voxel and MRS Block as fixed effects; Voxel × Block: *F*(1, 143) = 0.10, *p* = 0.74). That is, GABA increased significantly during training independent of Voxel (LME model for FD GABA with MRS Block as fixed effect; main effect of MRS Block: *F*(1, 145) = 6.13, *t* = 2.48, *p* = 0.01). In contrast, for the SN task GABA changes during training differed in the two regions (LME model for SN task with Voxel and MRS Block as fixed effects; Voxel × Block: *F*(1, 121) = 13.06, *p* = 0.0004). That is, we found a significant decrease for OCT GABA (LME model for SN task with MRS Block as fixed effect; main effect of Block: *F*(1,58) = 16.65, *t* = −4.08, *p* = 0.0001), but a nonsignificant increasing trend for PPC GABA (LME model for SN task with MRS Block as fixed effect; main effect of Block: *F*(1,63) = 3.26, *t* = 1.80, *p* = 0.08).

We conducted the following control analyses that corroborated our results (see Supplementary Methods for more details). First, we demonstrated that the learning-dependent changes we observed in GABA levels: (a) were not simply driven by GABA measurements at baseline, (b) could not be simply due to the order with which the MRS voxels were acquired during training. Second, we tested whether the learning-dependent changes we observed in GABA, were simply due to differences in data quality across multiple MRS measurements. In particular, we showed that there were no significant differences in the signal-to-noise ratio (SNR) of MRS measurements over time, nor BOLD effects on MRS spectra as represented by narrowing of the linewidth. In addition, we conducted a no-training control experiment on an independent group of participants (*n* = 8). We measured OCT GABA before and after exposing the participants to Glass pattern stimuli for a duration comparable to the training in our main study. During this time participants performed a fixation task rather than the SN or FD tasks. We did not observe any significant differences (*t*(7) = 1.56, *p* = 0.16) between two measurements of OCT GABA, suggesting that the GABA changes we observed in our main study were specific to task-training. Third, we showed that the learning-dependent changes we observed in GABA/tCr (a) could not be explained simply by changes in tCr concentration over time (Supplementary Figure 5), (b) remained significant when we referenced GABA to water rather than tCr concentration (Supplementary Figure 4), (c) were specific to GABA and did not generalize to Glutamate (Fig. 3b).

### Learning-dependent changes in GABA relate to behavior

**Fig. 4:**
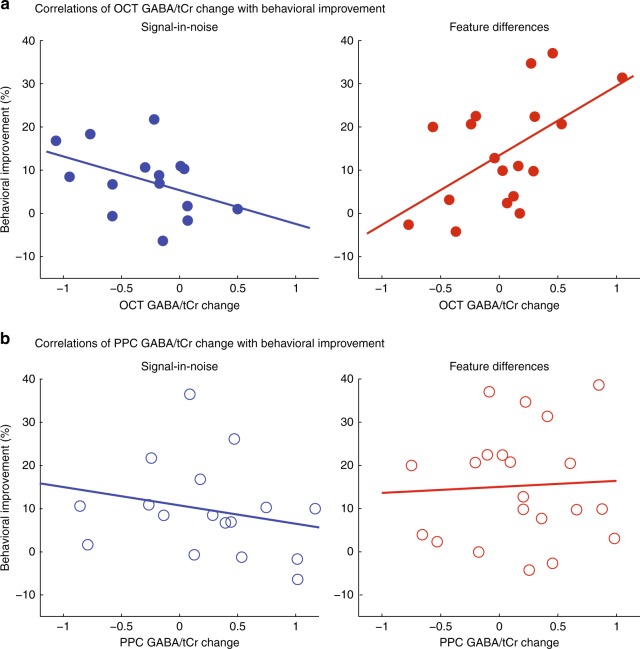
Correlating γ-aminobutyric acid (GABA) change with behavioral improvement. **a** Skipped Pearson’s correlations showing a significant negative correlation of GABA/tCr change in occipito-temporal cortex with behavioral improvement for the signal-in-noise task (*n* = 16, *r* = -0.43, CI = [–0.75, –0.002]), whereas a significant positive correlation for the feature differences task (*n* = 19, *r* = 0.55, CI = [0.10, 0.78]). We computed GABA/tCr change for each participant as the difference between GABA/tCr in the training block with maximum performance and GABA/tCr in the baseline block. Note that the temporal resolution of GABA measurements does not allow us to separate the different processes associated with different events in a trial (e.g., stimulus vs. response). Thus, the correlations we report here relate learning-dependent changes in GABA levels to learning-dependent changes in overall task performance. The plots indicate that for a small number of participants the data deviated from the overall pattern of the correlation; e.g., for some participants in the SD task, GABA/tCr values were higher rather than lower compared with baseline. Our treatment of the data (i.e., GABA data are expressed as percent over baseline; behavioral improvement is expressed as percent over early performance) accounts for potential differences across participants in performance early in training or baseline GABA before training. It is possible that this individual variability was due to the single training session employed in our study during which participant performance did not saturate (i.e., average 72% maximum performance across participants). **b** Correlations of posterior parietal GABA/tCr change from baseline with behavioral improvement were not significant for the signal-in-noise (*n* = 17, *r* = –0.23, CI = [–0.61, 0.19]) or the feature differences task (*n* = 21, *r* = 0.05, CI = [–0.37, 0.43]). Significant correlations are indicated by closed symbols; nonsignificant correlations by open symbols

We next tested whether the learning-dependent changes we observed in occipito-temporal and parietal GABA related to behavioral improvement. Correlating change in OCT GABA (see Supplementary Methods) to behavioral improvement showed differences between tasks. Specifically, we observed a significant negative correlation of OCT GABA change with behavioral improvement (*r* = −0.43, CI = [−0.75, −0.02]) for the SN task, whereas a significant positive correlation (*r* = 0.55, CI = [0.10, 0.78]) for the FD task (Fig. 4a). A linear regression analysis confirmed this dissociation (OCT GABA change × Task interaction: *F*(1, 29) = 9.03, *p* = 0.005), suggesting that lower vs. higher occipito-temporal GABA relates to improved performance when learning to detect targets vs. discriminate feature differences, respectively. This dissociable result between tasks cannot be simply explained by differences in task difficulty, as participants showed similar behavioral improvement across tasks (Fig. 1b).

In contrast to these correlations of OCT GABA change with behavioral improvement, we did not observe any significant correlations between PPC GABA change and behavioral improvement for either task (SN: *r* = −0.23, CI = [−0.61, 0.19]; FD: *r* = 0.05, CI = [−0.37, 0.43]) nor a significant PPC GABA change × Task interaction (*F*(1, 34) = 0.57, *p* = 0.45) (Fig. 4b). Following previous work on the role of the PPC early rather than later in training^35^, we next tested the link between PPC GABA and performance early in training for the two tasks. A linear regression model with baseline PPC GABA and Task as predictors of behavioral performance (first training block) showed a significant effect of PPC GABA (*F*(1, 37) = 4.69, *p* = 0.04), but no interaction between PPC GABA and Task (*F*(1, 37) = 0.03, *p* = 0.85). This result was confirmed by a significant positive correlation between baseline PPC GABA and performance in the first training block (*r* = 0.34, CI = [0.06, 0.59]), suggesting GABAergic inhibition in the parietal cortex before training relates to suppression of task-irrelevant information early in the training across tasks. We did not find a significant effect of OCT GABA (*F*(1, 33) = 2.14, *p* = 0.15) nor an interaction between OCT GABA and Task (*F*(1, 33) = 2.33, *p* = 0.14), suggesting that this result linking GABA to performance early in training was specific to parietal cortex.

### Functional connectivity at rest relates to behavior and GABA

Previous studies have shown that functional connectivity in motor^14^ and visual^36^ networks relates to behavioral improvement and learning-dependent plasticity. Further, GABAergic inhibition has been suggested to shape network connectivity^37^. In particular, extra-synaptic GABA has been shown to relate to local oscillatory activity in the high gamma frequency range^38^ and to inter-regional functional connectivity^39^. In the human brain, MRS-assessed GABA has been linked to functional connectivity as measured by resting-state fMRI^37,40,41^, suggesting that GABAergic inhibition may relate to local neural dynamics^42^. Here we test whether functional connectivity between the OCT and PPC relates to behavioral improvement and GABA changes during training to detect targets in clutter vs. discriminate fine features.

**Fig. 5:**
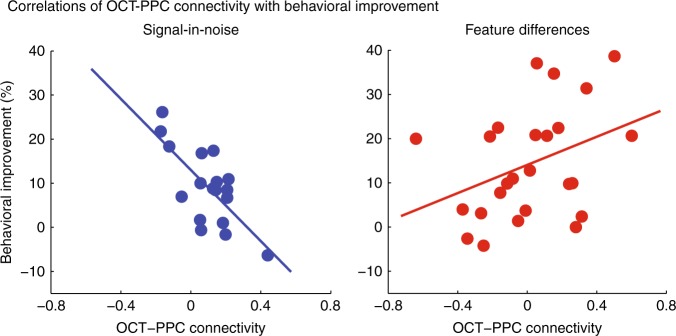
Correlating occipito-temporal cortex (OCT)-posterior parietal cortex (PPC) functional connectivity with behavioral improvement. Skipped Pearson’s correlations showing a significant negative correlation of OCT-PPC connectivity with behavioral improvement for the signal-in-noise task (*n* = 21, *r* = −0.72, CI = [−0.90, −0.29]), whereas a significant positive correlation for the feature differences task (*n* = 25, *r* = 0.37, CI = [0.03, 0.69])

First, we tested whether functional connectivity between these regions relates to behavioral improvement in each of the two learning tasks. We extracted the resting-state fMRI (rs-fMRI) time course only from the gray matter voxels within the OCT MRS voxel and the PPC MRS voxel (see Supplementary Methods). We measured functional connectivity by correlating the rs-fMRI time courses at rest between these two regions (OCT-PPC connectivity) (see Supplementary Methods). We observed a significant positive correlation between OCT-PPC connectivity and behavioral improvement for the FD task (*r* = 0.37, CI = [0.03, 0.69]), whereas a significant negative correlation for the SN task (*r* = −0.72, CI = [−0.90, −0.29]) (Fig. 5). This dissociation in the relationship of OCT-PPC connectivity and behavioral improvement between tasks was confirmed by a linear regression showing a significant interaction between OCT-PPC connectivity and Task (*F*(1, 39) = 10.72, *p* = 0.002), suggesting that higher connectivity between parietal and visual cortex facilitates learning of fine feature differences. We interpret these results according to previous studies that show both positive and negative values for resting-state connectivity (e.g.,^36^). Positive connectivity values (i.e., correlated signals) are typically interpreted to indicate integration or cooperation within or between brain regions. In contrast, negative connectivity values (i.e., anti-correlated signals) are typically interpreted to indicate segregation of processing or competition between networks. Thus, the correlations we observed between resting-state connectivity and behavioral improvement suggest higher cooperation between OCT and PPC for learning fine features differences (FD task) than learning to detect targets from noise (SN task). Further, to test whether this link between functional connectivity and behavioral improvement is specific to interactions between parietal and visual areas, we extracted rs-fMRI for two additional control regions: early visual cortex and motor cortex. We did not observe any significant results for correlations of behavioral improvement and rs-fMRI connectivity between OCT and (a) early visual cortex (SN: *r* = 0.37, CI = [–0.17, 0.70]; FD: *r* = –0.22, CI = [–0.56, 0.15]) or (b) motor cortex (SN: *r* = –0.26, CI = [–0.61, 0.12]; FD: *r* = –0.06, CI = [–0.39, 0.42]) (see Supplementary Methods).

**Fig. 6:**
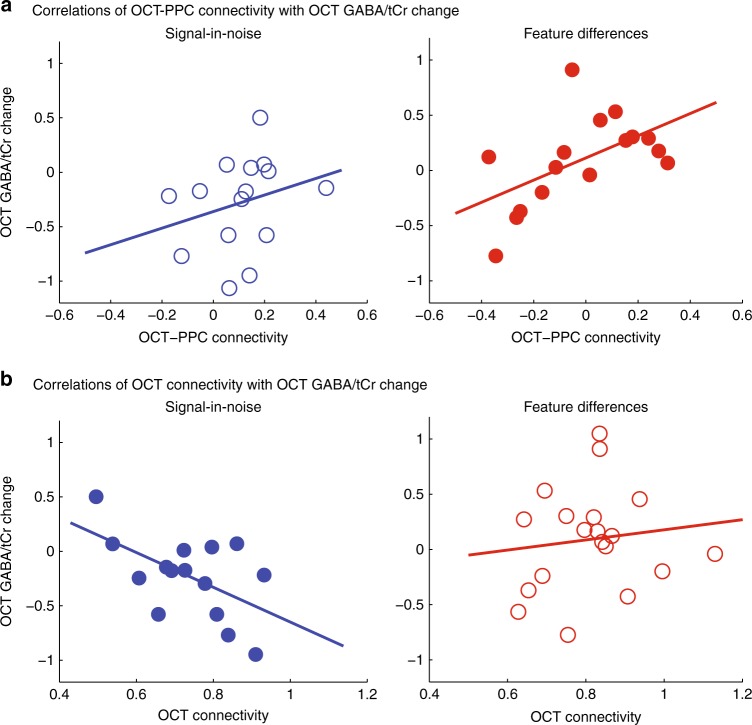
Correlating functional connectivity with occipito-temporal cortex (OCT) γ-aminobutyric acid (GABA)/tCr change. **a** Skipped Pearson’s correlations showing a significant positive correlation of OCT-posterior parietal cortex (PPC) connectivity with OCT GABA/tCr change for the feature differences task (*n* = 19, *r* = 0.55, CI = [0.13, 0.87]), but not the signal-in-noise task (*n* = 16, *r* = 0.27, CI = [−0.08, 0.60]). We computed GABA/tCr change for each participant as the difference between GABA/tCr in the training block with maximum performance and GABA/tCr in the baseline block. **b** Skipped Pearson’s correlations showing a significant negative correlation of functional connectivity within the occipito-temporal cortex with OCT GABA/tCr change for the signal-in-noise task (*n* = 16, *r* = −0.55, CI = [−0.86, −0.01]), but not the feature differences task (*n* = 19, *r* = 0.12, CI = [−0.29, 0.51]). Significant correlations are indicated by closed symbols; nonsignificant correlations by open symbols

Second, we tested whether OCT-PPC functional connectivity relates to changes in visual cortex GABA during training. Our results showed a significant positive correlation between functional connectivity and OCT GABA change for the FD task (*r* = 0.55, CI = [0.13, 0.87]), but not the SN task (*r* = 0.27, CI = [–0.08, 0.60]) (Fig. 6a). These correlations were specific to GABA changes in OCT. That is, there were no significant correlations between OCT-PPC connectivity and PPC GABA change for the FD task (*r* = 0.29, CI = [–0.37, 0.69]) nor the SN task (*r* = 0.20, CI = [–0.31, 0.60]). A multivariate linear regression showed that OCT-PPC connectivity had a significant effect on GABA change in OCT (*F*(1, 14) = 20.74, *p* = 0.0005), but not PPC (*F*(1, 14) = 0.46, *p* = 0.51) for the FD task. The correlation between functional connectivity and OCT GABA change for the FD task remained significant (*r* = 0.49, CI = [0.10, 0.82]) when we tested for percentage GABA change (GABA change/baseline GABA), suggesting that our results could not be due to differences in baseline GABA. These results show that parietal-visual cortex connectivity relates to GABAergic inhibition in visual cortex, suggesting top-down influences to suppressive processing in visual cortex for learning fine feature differences.

Interestingly, for the SN task we observed a significantly negative correlation between connectivity within the OCT (i.e., OCT connectivity: connectivity measured as rs-fMRI correlations across voxels within the OCT MRS voxel, see Supplementary Methods) and OCT GABA change (*r* = –0.55, CI = [–0.86, –0.01]) (Fig. 6b). That is, higher OCT connectivity related to decreased OCT GABA with training, suggesting that learning to detect targets in clutter is supported by local connectivity and decreased GABAergic inhibition within visual cortex. Correlations of functional connectivity and GABA change in the OCT were not significant for the FD task (*r* = 0.12, CI = [–0.29, 0.51]) and were significantly different from correlations for the SN task (*Z* = 2.33, *p* = 0.02). The correlation between OCT connectivity and GABA change for the SN task remained significant (*r* = –0.55, CI = [–0.88, –0.003]), when we used percentage GABA change (GABA change/baseline GABA), suggesting that our results could not be due to differences in baseline GABA. Correlating OCT connectivity with PPC GABA change did not show any significant results (*r* = 0.18, CI = [–0.39, 0.60]), suggesting that local connectivity relates specifically to GABA changes in visual cortex.

Taken together, our results suggest that learning fine discriminations involves interactions between decision-related (posterior parietal) and sensory (visual) areas that may facilitate retuning of task-relevant features in visual cortex. In contrast, learning to detect targets from clutter involves local processing and decreased inhibition in visual cortex. To further test this proposal, we performed moderation analyses^43^ that allowed us to test whether the influence that an independent variable (i.e., GABA change) has on the outcome (i.e., behavioral improvement) is moderated by a moderator variable (i.e., connectivity). Our results showed that OCT-PPC connectivity moderates the relationship between OCT GABA and behavior for the FD task (*F*(1, 15) = 6.19, *p* = 0.03) but not the SN task (*F*(1, 12) = 0.74, *p* = 0.41). In contrast, local connectivity within OCT moderates the relationship between GABA change and behavior for the SN task (*F*(1, 12) = 7.65, *p* = 0.02) but not the FD task (*F*(1, 15) = 2.80, *p* = 0.11). These moderation analyses suggest that the relationship between learning-dependent changes in GABA and behavior is moderated by functional connectivity; that is, interactions between parietal and visual areas for the FD task, whereas local connectivity within visual cortex for the SN task.

## Discussion

Here, we provide evidence that learning improves perceptual decisions (i.e., learning to detect targets in clutter or discriminate highly similar features) by suppressive interactions in the human brain. First, we demonstrate dissociable GABAergic inhibition mechanisms for learning in a posterior cortical network (i.e., occipito-temporal and posterior-parietal cortex) known to be involved in perceptual decisions. In particular, increased GABAergic inhibition in the PPC with training suggests suppressive processing of task-irrelevant information. In contrast, changes in occipito-temporal GABA with training relate to enhanced target detection and discriminability, suggesting learning-dependent changes in the processing of behaviorally relevant features. Second, we provide evidence that interactions within this network, as measured by functional brain connectivity at rest, gate suppressive processing of sensory signals. Learning to detect targets from clutter involves local processing and disinhibition in visual cortex, while learning feature differences involves suppressive interactions between decision-related (parietal) and visual areas.

Previous studies have used MRS to measure GABA in the context of visual^15^, sensory-motor^44–46^, and learning tasks^47,48^. Here, we exploited the high spectral resolution afforded by ultra-high field (7T) MRS to reliably resolve GABA from other metabolites^19,20^ and measure GABA changes over time at faster time scales than typically recorded at lower field strength. However, as the temporal resolution of MRS-assessed GABA is in the order of minutes, it is not possible to separate GABAergic inhibition related to different processes within a trial (i.e., stimulus processing, perceptual judgment, response, feedback); thus, it is likely that the observed GABA changes relate to aggregate signals across all processes involved in the tasks performed by the participants. Despite this, our methodology provides two main advantages over previous work. First, moving beyond the standard correlational approach that relates single measurements of GABA at baseline to behavior, we implemented an interventional approach to test whether GABAergic inhibition changes due to task-specific training. In particular, we captured learning-dependent changes in functional GABA during training. Two previous studies reporting measurements of GABA during training have focused on motor cortex GABAergic inhibition during training on a motor learning task^13,49^. Second, we implemented an MRS protocol that allows us to track longitudinal changes in GABA during training from both OCT and PPC that are thought to play distinct functional roles in perceptual learning^29,30^. Using this protocol, in combination with functional connectivity as measured by rs-fMRI, we tested for the role of suppressive interactions between sensory and decision-related areas in perceptual learning.

Our findings provide evidence for distinct GABAergic inhibition mechanisms in PPC vs. OCT for learning. In particular, we demonstrate a common mechanism across tasks in the PPC. That is, GABA increases during training may facilitate suppression of task-irrelevant information (i.e., background clutter when learning to detect targets, task-irrelevant features when learning fine differences). It is unlikely that changes in GABA levels with training reflect reduced attention to the task, as the task remained sufficiently demanding (i.e., mean maximum performance was 72%) during the single training session employed in our study. Our findings are consistent with the known role of parietal cortex in perceptual decision-making and attentional selection. In particular, the PPC has been implicated in detecting low-saliency targets by suppressing distractors^50^, providing a salient representation of the environment and top-down attentional feedback^51^, accumulating sensory information^52^ and directing attention to task-relevant features^29,51^. Interestingly, we show that parietal cortex GABA before training relates to performance early in training across tasks. This is in contrast with visual cortex changes in GABA that relate to improvement in behavioral performance during training. This finding suggests that suppression of task-irrelevant information in the PPC may precede suppression in the visual cortex related to the processing of task-relevant features. This is consistent with previous studies showing that transcranial magnetic stimulation (TMS) in the parietal cortex disrupts performance in visual discrimination tasks early in the training compared with TMS in the visual cortex that disrupts performance after training^35^.

In contrast to this common GABAergic inhibition mechanism across tasks in the PPC, our results in the visual cortex suggest distinct GABAergic mechanisms between tasks that relate to improved behavioral performance. In particular, decreased occipito-temporal GABA related to improved performance when training to detect targets in clutter, while increased occipito-temporal GABA related to improved performance when training to discriminate fine feature differences. These results suggest that learning to detect targets in clutter is implemented by decreased local GABAergic inhibition that may facilitate noise filtering and feature detection. Disinhibition of the visual cortex may facilitate cortical recruitment that enhances probability summation and SNR for target detection^53^. This mechanism is consistent with previous animal studies linking GABAergic inhibition to neural gain^25^ and interventional studies showing that blocking GABAergic inhibition increases neural gain^24^. In contrast, discriminating highly similar targets is implemented by increased GABAergic inhibition in the visual cortex that may facilitate learning through feature template retuning. This mechanism is consistent with neurophysiological studies linking GABAergic inhibition to cortical tuning^26^ and pharmacological interventions showing that GABA agonists enhance orientation selectivity in visual cortex^27,28^, whereas blocking GABAergic inhibition results in broader neural tuning^27^. Interestingly, recent work has implicated different populations of inhibitory interneurons in neural gain vs. tuning^54^ that may contribute differentially to learning by noise filtering vs. feature template retuning. In particular somatostatin (SOM)-positive interneurons have been implicated in spatial summation^55^ and have been shown to gate plasticity by providing contextual information^56^. In contrast, parvalbumin (PV)-positive interneurons have been implicated in selective inhibition^16^ that sharpens feature representations after training^57^. It is therefore possible that SOM interneurons may support learning to detect targets from clutter (SN task) through noise filtering, whereas PV interneurons may support learning fine differences (FD task) through feature retuning. Thus, our findings propose testable hypotheses for further animal studies on the micro-circuits that mediate adaptive behavior and underlie the macroscopic learning-dependent plasticity as measured by human brain imaging.

We interpret these links between MRS-assessed GABA, behavior and possible neural mechanisms with caution, as the precise mechanisms that underlie changes in GABA as measured by MRS remain under investigation. In particular, it is unclear whether MRS-assessed GABA represents the entire pool of GABA available in a voxel. It is possible that the individual GABA pools are not equally visible using MRS^13,58^. Another possibility is that MRS-assessed GABA reflects the exchange between the intra-cellular and synaptic pools of GABA^59^. A number of studies using paired-pulse TMS (e.g., ^60,61^) have shown that MRS-assessed GABA does not relate to GABA_A_ or GABA_B_ activity and therefore it is unlikely to reflect synaptic transmission of GABA. It is more likely that MRS-assessed GABA reflects ambient extracellular GABA that contributes to tonic GABAergic activity (for reviews e.g., ^62,63^). This is consistent with animal studies showing that GABA synthesis^64^ is associated with Glutamic Acid Decarboxylase (GAD)_67_ that is predominantly found throughout the cell, rather than GAD_65_ that is found in axon terminals^65^. Finally, we did not observe changes in glutamate simultaneously with the change in GABA observed during training. Thus, it is unlikely that the changes in GABA reflect overall changes in the metabolite cycling. GABA undergoes rapid turnover in the mammalian cortex, and GAD activity has been shown to be rapidly modulated in a variety of physiological processes^66^ in both human^67^ and animal^68^ studies. Future studies combining invasive investigations in animals (e.g., two photon imaging of interneurons) with non-invasive MRS are necessary to shed more light on the basis MRS-assessed GABA.

Finally, investigating functional connectivity within this posterior cortical network revealed suppressive interactions that relate to our ability to improve perceptual judgments through training. We demonstrate that higher connectivity between parietal and visual cortex at rest relates to increased GABA levels in visual cortex during training and behavioral improvement in fine feature discrimination. This finding suggests top-down influences from the parietal cortex on suppressive processing in the visual cortex for retuning feature templates. Previous theoretical investigations have proposed that sensory selectivity is enhanced by suppressive feedback mechanisms that change recurrent processing in visual cortex^23^ and relate to attention-guided selection of behaviorally relevant information^69^. Our findings suggest that suppression of task-irrelevant information in the parietal cortex enhances tuning of task-relevant features in visual cortex through top-down feedback, consistent with previous proposals that perceptual learning re-weights sensory processing^30,31^. In contrast, we show that higher connectivity within the visual cortex (rather than connectivity between visual and parietal cortex) relates to decreased GABA in visual cortex and behavioral improvement in detecting targets from clutter. Lateral interactions within the early visual cortex are shown to support contextual processing and shape integration^53,70^. Further, recurrent processing has been implicated in robust representations of ambiguous stimuli in higher visual cortex^71^, suggesting that local connectivity in occipito-temporal cortex facilitates visual processing under uncertainty.

In sum, our findings provide evidence for distinct GABAergic inhibition mechanisms that support our ability to optimize perceptual decisions through training. We propose that local suppressive processing within visual cortex enhances target detection, whereas top-down suppression from decision-related areas (i.e., posterior parietal cortex) enhances retuning of task-relevant features for fine discrimination in the visual cortex. Decision-making models have proposed suppressive mechanisms that resolve competition between neuronal ensembles that represent behavioral choices^72^. Our findings provide novel insights in understanding how these suppressive mechanisms are implemented in decision-related and sensory areas in the human brain and optimize our ability for perceptual decisions through training.

## Methods

### Participants

Fifty participants (32 females; mean age 25.6 ± 3.3 years) took part in this study. Data from three participants were not useable due to technical problems. All participants were right handed, had normal or corrected-to-normal vision, and gave written informed consent. The study was approved the University of Oxford Central University Research Ethics committee (MSD-IDREC-C1-2014-081).

### Stimuli

We presented participants with Glass patterns generated using previously described methods^33,34^. In particular, stimuli were defined by white dot pairs (dipoles) displayed within a square aperture on a black background. Experiment stimuli (size = 7.9^o^ × 7.9^o^) were presented in the center of the screen. The dot density was 3%, and the Glass shift (i.e., the distance between two dots in a dipole) was 16.2 arc min. The size of each dot was 2.3 × 2.3 arc min^2^. For each dot dipole, the spiral angle was defined as the angle between the dot dipole orientation and the radius from the center of the dipole to the center of the stimulus aperture. Each stimulus comprised dot dipoles that were aligned according to the specified spiral angle (signal dipoles) for a given stimulus and noise dipoles for which the spiral angle was randomly selected. The proportion of signal dipoles defined the stimulus signal level.

We generated radial (0^o^ spiral angle) and concentric (90^o^ spiral angle) Glass patterns by placing dipoles orthogonally (radial stimuli) or tangentially (concentric stimuli) to the circumference of a circle centered on the fixation dot. Further, we generated intermediate patterns between these two Glass pattern types by parametrically varying the spiral angle of the pattern from 0° (radial pattern) to 90° (concentric pattern). We randomized the presentation of clockwise (0° to 90° spiral angle) and counterclockwise patterns (0° to –90° spiral angle) across participants. A new pattern was generated for each stimulus presented in a trial, resulting in stimuli that were locally jittered in their position.

For the SN task, radial and concentric stimuli (spiral angle: 0° ± 90°) were presented at 24% ± 1% signal level; that is, 76% of the dipoles were presented at random position and orientation. For the FD task, stimuli were presented at 100% signal and spiral angle of ± 38^o^ (radial) or ± 52^o^ (concentric) (Fig. 1a). To control for stimulus-specific training effects, we presented each participant with a newly generated set of stimuli. To control for potential local adaptation due to stimulus repetition, we jittered ( ± 1–3°) the spiral angle across stimuli. These procedures ensured that learning related to global shape rather than local stimulus features.

### Experiment design

All participants took part in a single brain imaging session (Fig. 2a) during which they were randomly assigned and trained on either the SN or the FD task. We recorded whole-brain rs-fMRI data before training while participants fixated on a cross at the center for the screen.

Following the rs-fMRI scan, we recorded MRS GABA before and during training (Fig. 2a). We collected MRS GABA from one baseline block before training and three blocks during task training. Each block comprised two MRS acquisitions: one from OCT and one from PPC. The order of the voxels within each block was counterbalanced across participants. During each training block, participants were presented with stimuli for 400 trials (200 trials per MRS voxel acquisition). During the baseline MRS block (400 trials) participants engaged in a task with similar stimuli as those presented during the training; that is, participants viewed random dot patterns (0% signal dipoles) and were asked to respond (button press) as soon as a pattern appeared. This ensured that differences in GABA between blocks could not be simply attributed to differences in overall alertness. During the MRS training blocks (3 MRS blocks, 400 trials each), participants were presented with Glass patterns and were asked to judge and indicate by button press whether the presented stimulus in each trial was radial or concentric. Two stimulus conditions (radial vs. concentric Glass patterns; 200 trials per condition), were presented for each training block. For each trial, a stimulus was presented for 300 ms and was followed by fixation (i.e., blank screen with a central fixation dot) while waiting for the participant’s response (self-paced training paradigm). Trial-by-trial feedback was provided by means of a visual cue (green tick for correct, red ‘x’ for incorrect) followed by a fixation dot for 500 ms before the onset of the next trial. Average time to complete a trial ranged between 1.4 s and 1.9s  across participants (1.66 ± 0.16 s for SN; 1.69 ± 0.16 s for FD). Mean trial duration across participants decreased across MRS blocks for both tasks (LME model for Trial duration with Task and MRS Block as fixed effects; main effect of Block: *F*(1, 113) = 4.68, *p* = 0.03) but did not differ between tasks (main effect of Task: *F*(1, 113) = 0.12, *p* = 0.73; Task × Block: *F*(1, 113) = 0.002, *p* = 0.97), suggesting that participant became faster in their judgments with training for both tasks. Each MRS acquisition lasted for 5 min and 56 s, and each MRS training block (400 trials) for 11 min and 11 s ± 63 s. Thus, in most cases training was completed within the duration of the training block (i.e., 2 MRS acquisitions × 5 min 56 s). In the event that the training took longer than the MRS block, the next MRS acquisition was delayed until completion of the previous training block.

### Data acquisition

Experiments were conducted at the Wellcome Centre for Integrative Neuroimaging, using a Siemens 7T Magnetom (Siemens, Erlangen) with a 32-channel head coil.

We acquired structural data (MPRAGE; TR 2200 ms; TE 2.82 ms; slice thickness 1.0 mm; in-plane resolution 1.0 × 1.0 mm^2^; GRAPPA factor = 4) and echo planar imaging data (gradient echo-pulse sequences) from 40 slices (TR 2250 ms; TE 28 ms; slice thickness 3.0 mm; in-plane resolution 3.0 × 3.0 mm^2^; GRAPPA factor = 2, 140 volumes).

We acquired MRS data using a semi-localization by adiabatic selective refocusing (semi-LASER) sequence^73^ (64 averages, TR 5010 ms, TE 36 ms). We measured two MRS voxels (2 × 2 × 2 cm^3^ isotropic), one in the left OCT (OCT voxel) and one in the left PPC (PPC voxel), avoiding contact with the dura to minimize macromolecule contamination. We focused on the left hemisphere as previous fMRI^50^ and TMS^35^ studies have shown that the left PPC is involved in suppressing distracting signals. To cover both areas with the same dielectric pad, we placed both MRS voxels on the left hemisphere.

### Statistical analyses

To compare changes in behavioral performance and neurotransmitter concentrations across blocks, tasks, and MRS voxels, we used a LME approach. LME models allow modeling of longitudinal data (i.e., multiple measurements over time) and can account for missing values across participants. In each of the models, we tested both for random (Participants) and fixed (MRS Block, Voxel, and Task) effects. To relate behavioral improvement to GABA changes and rs-fMRI connectivity, we computed Pearson skipped correlations using the Robust Correlation Toolbox^74^. This method accounts for potential bivariate outliers and determines statistical significance using bootstrapped confidence intervals (CI) for 1000 permutations. Note that bivariate outliers are not shown in the data figures. To directly compare the relationship of GABA change and rs-fMRI connectivity with behavioral improvement between the two tasks, we used linear regression models with interaction terms. Data distribution assumptions of normality and heteroscedasticity of variance were verified using Shapiro–Wilk and Levene’s tests, respectively. All statistical tests are two sided.

## Supplementary Information

Supplementary Information

## Data Availability

Data files have been uploaded on the Cambridge Data Repository: 10.17863/CAM.33382.
