# Supplementary material for: Learning to optimize perceptual decisions through suppressive interactions in the human brain
Source: Nat Commun. 2019 Jan 28;10:474. doi: 10.1038/s41467-019-08313-y (PMC6349878; doi:10.1038/s41467-019-08313-y)
Supplement: Supplementary file 1 — Supplementary Information [file 41467_2019_8313_MOESM1_ESM.pdf]

Supplementary Information

Frangou et al., Learning to optimize perceptual decisions through suppressive interactions in the human brain.

Supplementary Methods

MRS acquisition

We chose to utilize a short-echo, full signal intensity semi-LASER sequence to achieve lower apparent T2 relaxation, minimal J-coupling evolution and smaller chemical shift displacement errors relative to the PRESS and STEAM sequences [1]. In addition, the adiabatic refocusing pulses in the semi-LASER provided minimal signal loss, high B1+ insensitivity and localization against the varying destructive interferences throughout the brain at ultra-high field. This MRS sequence has been extensively tested and resulted in high quality spectra (Supplementary Figures 1,2,3) across high and ultra-high field magnetic fields at different MRI centers [1–5]. We used VAPOR [6] water suppression and outer volume suppression [5].

A dielectric pad (BaTiO3, 14.5 x 12.5 cm^2) was placed over the left occipito-parietal cortex to increase B1 efficiency in the regions where the MRS voxels were placed [7]. First and second order shims were adjusted for each voxel separately using FASTMAP (fast, automatic shimming technique by mapping along projections) with echo-planar imaging readout [8]. Acquisition parameters were optimized for each voxel by determining the appropriate transmit voltage (flip angle calibration) and flip angle (VAPOR calibration), to maximize readout and water suppression respectively [7].

Two non-suppressed water spectra were acquired: one for eddy current correction and reconstruction of the phased array spectra (the RF pulses of the VAPOR scheme were turned off, NT = 2, TR = 5.010 s, TE = 36 ms, number of dummy scans = 2, spectral bandwidth = 6 kHz, data points = 2048) and one for use as reference for metabolite quantification (VAPOR and OVS schemes turned off in order to eliminate magnetization transfer effects, NT = 2, TR = 5.010 s, TE = 36 ms, number of dummy scans = 2, spectral bandwidth = 6 kHz, data points

= 2048). The reconstruction of the phased array spectra included weighting the spectra based on the sensitivity of each receive element at the VOI and correcting for the different constant phase shift terms of the complex spectra prior to the summation. Single scan spectra summed from 32 channels were corrected for frequency and phase variations induced by subject motion and then summed before further analyses.

Data analysis

Behavioral data analysis

To take into account individual variability in performance, we estimated behavioral improvement as the difference in mean performance (i.e. mean accuracy per 200 trials) between the first training block and the training block with maximum performance per participant (85% of the participants achieved maximum performance during the last two MRS measurements), divided by performance in the first training block.

MRS data analysis

Eddy-current correction and reconstruction of the phased array spectra was applied using in-house scripts. Water residual signal was removed using a Hankel singular value decomposition (HLSVD) MATLAB routine [9]. LC-Model [10] was used to quantify metabolite concentrations in the range of 0.5 to 4.2 ppm (Fig. 2c, Supplementary Figures 1,2) using optimal initialization parameters.

The model spectra of aspartate (Asp), ascorbate/vitamin C (Asc), glycerophosphocholine (GPC), phosphocholine (PC), creatine (Cr), phosphocreatine (PCr), γ-amino-butyric acid (GABA), glucose (Glc), glutamine (Gln), Glutamate (Glu), glutathione (GSH), myo-inositol (myo-Ins), N acetylaspartate (NAA), Nacetylaspartylglutamate (NAAG), phosphoethanolamine (PE), scyllo-inositol (scyllo-Ins) and taurine (Tau) were generated

based on previously reported chemical shifts and coupling constants by using GAMMA/PyGAMMA simulation library of VESPA (Versatile Simulation, Pulses and Analysis) for carrying out the density matrix formalism. Simulations were performed with the same RF pulses and sequence timings as that on the 7T system in use [11].

We followed the same macromolecule inclusion procedure as Bednarik et al.[12]. Macromolecule spectra acquired from the occipital cortex from 3 healthy volunteers, using an inversion recovery sequence (TR=3 s, TE=36 ms, inversion time TI=0.685 s), were included in the LCModel basis set. The residual signal of the methylene of tCr at 3.93 ppm was removed by post processing and the high-frequency noise was suppressed using a Gaussian filter (σ=0.05 s) before including the macromolecule spectrum into the LCModel basis set.

We referenced metabolite concentrations to the sum of the concentrations of Creatine (Cr) and Phosphocreatine (PCr), that is total Creatine (tCr). In particular, for each MRS voxel, we normalized GABA/tCr in each training block to GABA/tCr in the baseline block (Fig. 3). We computed GABA/tCr change for each participant as the difference between GABA/tCr in the training block with maximum performance and GABA/tCr in the baseline block. We chose tCr as reference for two main reasons. First, tCr concentration was measured in the same spectrum, concurrently with GABA, while water concentration was estimated from a different scan. Using another metabolite acquired in the same spectrum as reference accounts for the possibility of small but relevant changes in neuronal density and spectral data quality that might be expected during periods of task activity [13]. Second, referencing metabolites to tCr has been shown to have better reproducibility compared to other referencing methods [14] and has been widely used as a reference metabolite in MRS studies [15,16]. Our control analysis (see *'Control analyses')* confirmed that tCr concentration did not change significantly during training across voxels or tasks, suggesting that our results are specific to GABA changes and are not driven by changes in tCr concentration. Finally, we replicated our findings

(Supplementary Figure 4) using absolute GABA quantification (GABA referenced to water) to ensure that our results were not driven by the chosen reference (e.g. [3,17,18]).

Only data without lipid contamination, GABA CRLB values smaller than one standard deviation above the mean and GABA values per block within two standard deviations from the mean across participants were included in further steps of MRS related analyses. That is, OCT data for 6 participants (2 for SN, 4 for FD) and PPC data for 6 participants (4 for SN, 2 for FD) were excluded due to high CRLB values. Thus, data from 18 participants were included for further analysis for the SN and 22 participants for the FD task. To account for variability in tissue composition within the MRS voxel across participants, we conducted whole brain tissue-type segmentation of the T1-weighted anatomical scan using SPM12.2 (SPM segment) and calculated percentage of gray matter (GM) and white matter (WM) voxels in each of the MRS voxels.

rs-fMRI data pre-processing

We pre-processed the resting-state fMRI (rs-fMRI) data using SPM12.2 (http://www.fil.ion.ucl.ac.uk/spm/software/spm12/) following the optimized pipeline described in recent work [19]. Data were excluded from one participant with incomplete data acquisition. We first processed the T1-weighted anatomical images by applying brain extraction and segmentation (SPM segment). From the segmented T1 we created a white matter (WM) mask and a cerebrospinal fluid (CSF) mask. For each participant, we corrected the EPI data for slice scan timing (i.e. to remove time shifts in slice acquisition, SPM slice timing), motion (least squares correction) and susceptibility distortions (applying fieldmap correction, SPM realign & unwarp). We then co-registered the EPI data to the T1 image (rigid body) per participant and calculated the mean CSF and WM signal per volume (SPM coregister & reslice). We subsequently aligned the T1 image to the MNI space (affine) and

applied the same transformation to the EPI data and the MRS voxels (OCT, PPC) (SPM normalise). We resliced the aligned EPI data to native resolution (3 x 3 x 3 mm^3) and applied spatial smoothing with a 5mm isotropic full width at half maximum (FWHM) Gaussian kernel (SPM smooth). Finally, we despiked any secondary motion artifacts using the Brain Wavelet Toolbox v1.1 [20].

We modeled the pre-processed data in a first-level analysis model (SPM first-level analysis) using an autoregressive AR(1) model to treat for serial correlations and regressing out the signal from CSF, WM, the motion parameters (translation, rotation and their squares and derivatives) and the signal from noise components (i.e. components overlapping with ventricles or brainstem) [21].

Functional connectivity analysis

We computed functional connectivity measures (connectivity between MRS voxels, temporal coherence within each MRS voxel) based on the following method. We computed the overlap across participant MRS voxels for OCT and PPC separately and created a group MRS mask that included gray matter voxels present in at least 50% of the participants' MRS voxels. For each participant, we extracted the average time course of the gray matter voxels within each MRS mask. We then applied a 5th order Butterworth band-pass filter, between 0.01 and 0.08 Hz, to remove effects of scanner noise and physiological signals (respiration, heart beat) [22].

We computed the functional connectivity between the OCT and the PPC MRS voxels as the Pearson correlation between the average time course from each of the MRS masks. We then applied Fisher z-transform to the correlation coefficient and derived an OCT-PPC connectivity value per participant. To confirm the specificity of the OCT-PPC connectivity, we computed the functional connectivity between OCT and two control areas (V1, M1). We defined masks of equal size to the MRS masks based on anatomical co-ordinates: primary visual cortex (V1, MNI coordinates [3, -85, 5]) and left M1 (MNI coordinates [-39, -22, 53]).

We assessed the temporal coherence within each MRS mask (OCT, PPC) by correlating the time course of each voxel within the mask with the MRS mask's average time course. This method was first described by Van Dijk et al [23] and has been extensively used in recent studies [24–27]. We then applied Fisher z-transform to the correlation matrix and averaged the z-values across voxels, resulting in one connectivity value per participant and MRS voxel.

MRS Data quality controls

We provide data quality metrics and statistics showing that data quality is highly similar across MRS voxels and participant groups. In particular, we considered: a) differences in signal-to-noise ratio (SNR) across MRS measurements, b) BOLD effects on MRS spectra as indicated by narrowing of the linewidth, c) frequency drift, d) GABA CRLB values

First, we tested whether the learning-dependent changes we observed in GABA, were due to differences in SNR across MRS measurements potentially due to artifacts (e.g. head movement, gradient heating). For OCT GABA, there was no significant differences in SNR across blocks, nor a significant interaction between Task x block (LME model for OCT SNR with Task and MRS Block as fixed effects; main effect of Block: $F(1,118)=0.12$, $p=0.73$; Task x Block: $F(1,118)=0.45$, $p=0.50$). Similarly for PPC GABA, there was no significant effect of block, nor a significant interaction of Task x block (LME model for PPC SNR with Task and MRS Block as fixed effects; main effect of Block: $F(1,145)=0.52$, $p=0.47$; Task x Block: $F(1,145)=0.90$, $p=0.34$), suggesting that our results could not be explained simply by differences in MRS SNR over time.

Second, we tested whether the learning-dependent changes we observed in GABA, were simply due to BOLD effects. BOLD effects on MRS spectra are presented as narrowing of the linewidth [12,28]. To control for potential effects from BOLD on the GABA measurements, we compared spectral FWHM across MRS blocks for the two regions. For OCT GABA, there

was no significant differences in FWHM across blocks, nor a significant interaction between Task x block (LME model for OCT FWHM with Task and MRS Block as fixed effects; main effect of Block: $F(1,118)=1.28$, $p=0.26$; Task x Block: $F(1,118)=2.31$, $p=0.13$). Similarly for PPC GABA, there was no significant effect of block, nor a significant interaction of Task x block (LME model for PPC FWHM with Task and MRS Block as fixed effects; main effect of Block: $F(1,145)=2.77$, $p=0.10$; Task x Block: $F(1,145)=0.89$, $p=0.35$), suggesting that our results could not be explained simply by differences in peak linewidth over time.

Third, we calculated frequency drift across MRS spectra and found no systematic frequency drift due to the hardware instability across participants and measurements. Overall, frequency drift was below 5Hz (0.4 ± 1.5 Hz for OCT; -0.08 ± 1.9 Hz for PPC), suggesting minimal potential artefacts originating from system instability or head movement.

Finally, we used the CRLB provided by LCModel as the measure for quality of the fit of individual resonances and used metabolite concentration values with GABA CRLB values smaller than one standard deviation above the mean. OCT data for 6 participants (2 for SN, 4 for FD) and PPC data for 6 participants (4 for SN, 2 for FD) were excluded due to high CRLB values. Given the consistency in SNR and FWHM across tasks and MRS blocks, higher CRLBs observed during training could be attributed to reduction in GABA signal in the voxel [3,29], while decreased CRLBs are expected for increased GABA/tCr [30]. This link between CRLB and GABA values which has been reported in previous studies [3,29,30] suggests that it is not appropriate to reject spectra due to changes in CRLBs over time.

Control analyses

We conducted the following control analyses that corroborated our results showing mean changes in GABA with training and correlations with behavioral improvement.

First, to ensure our results were not simply driven by GABA measurements at baseline, we tested a linear mixed effects model on the training blocks only (i.e. excluding the baseline block; LME model for OCT GABA with Task and training MRS Block as fixed effects). This analysis showed a significant interaction between Task and MRS blocks (Task x Block: $F(1,83)=4.97$, $p=0.03$) and a significant main effect of MRS block ($F(1,83)=4.06$, $p=0.05$). These analyses suggest that the learning-dependent GABA changes we observed were due to training rather than simply differences in GABA between the training blocks and the baseline.

Second, we demonstrated that the learning-dependent changes we observed in GABA levels could not be simply due to the order with which the MRS voxels were acquired during training. For OCT GABA, the Task x Block interaction remained significant (Task x Block: $F(1,115)=13.51$, $p=0.0004$) when we included the order of voxel acquisition in the LME model . Further, there was no significant effect of MRS acquisition order (LME model for OCT GABA with Acquisition Order, Task and MRS Block as fixed effects; main effect of Order: $F(1,115)=0.08$, $p=0.78$; Order x Task x Block: $F(1,115)=1.58$, $p=0.21$) Similarly, for PPC GABA, the main effect of block remained significant ($F(1,141)=7.72$, $p=0.01$) and there was no significant effect of MRS acquisition order (LME model for OCT GABA with Acquisition Order, Task and MRS Block as fixed effects; main effect of Order: $F(1,141)=1.51$, $p=0.22$; Order x Task x Block: $F(1,141)=1.50$, $p=0.22$).

Third, we tested whether the learning-dependent changes we observed in GABA/tCr were driven by changes in tCr concentration during training. For OCT GABA, there were no significant differences in tCr across blocks, nor a significant interaction between Task x block (LME model for OCT tCr with Task and MRS Block as fixed effects; main effect of Block: $F(1,119)=0.11$, $p=0.74$; Task x Block: $F(1,119)=0.02$, $p=0.90$). Similarly for PPC GABA, there was no significant effect of block, nor a significant interaction of Task x block (LME

model for PPC tCr with Task and MRS Block as fixed effects; main effect of Block: $F(1,137)=0.59$, $p=0.45$; Task x Block: $F(1,137)=0.42$, $p=0.52$), suggesting that our results could not be explained simply by changes in tCr concentration over time (Supplementary figure 5).

Fourth, our results remained significant when we referenced GABA to water rather than tCr concentration (Supplementary figure 4), suggesting that our results replicate across referencing methods. That is, we observed a significant Task x block interaction for OCT (LME model for OCT GABA with Task and MRS Block as fixed effects; Task x Block: $F(1,119)=10.68$, $p=0.001$) and a significant main effect of block for PPC (LME model for PPC GABA with Task and MRS Block as fixed effects main effect of Block: $F(1,137)=7.08$, $p=0.01$).

Fifth, we tested whether the correlations we observed between GABA change and behavioral improvement were confounded by baseline GABA or variability in tissue composition across participants. The linear regression analysis showing the dissociable effect of OCT GABA changes on behavioral improvement between the two tasks (i.e.: OCT GABA change x Task Interaction: $F(1,29)=9.03$, $p=0.005$) remained significant when we used percentage OCT GABA change (OCT GABA change / OCT baseline GABA), suggesting that our results could not be due to differences in baseline OCT GABA. The same interaction remained significant a) when we controlled for tissue (GM+WM) composition ($F(1,29)=5.24$, $p=0.03$) and b) when we used partial volume corrected [31] and water scaled [31] GABA values ($F(1,29)=12.32$, $p=0.001$), suggesting that our results could not be due to variability in tissue composition across participants. Following previous studies, we used T2=46ms for water [32] and T2=100ms for all metabolites [33] for LCModel parameters ATTH2O and ATTMET, respectively.

Finally, to investigate the neurochemical specificity of our results, we tested for changes in Glutamate, the other major cortical neurotransmitter, during training (Fig. 3b). We found no significant differences in Glutamate changes between tasks (LME model for OCT Glutamate with Task and MRS Block as fixed effects Task x Block: $F(1,119)=0.53$, $p=0.47$; for PPC Glutamate: main effect of Block: $F(1,137)=2.65$, $p=0.11$), suggesting that our results were specific to GABA and do not generalize to Glutamate.

Supplementary Figures

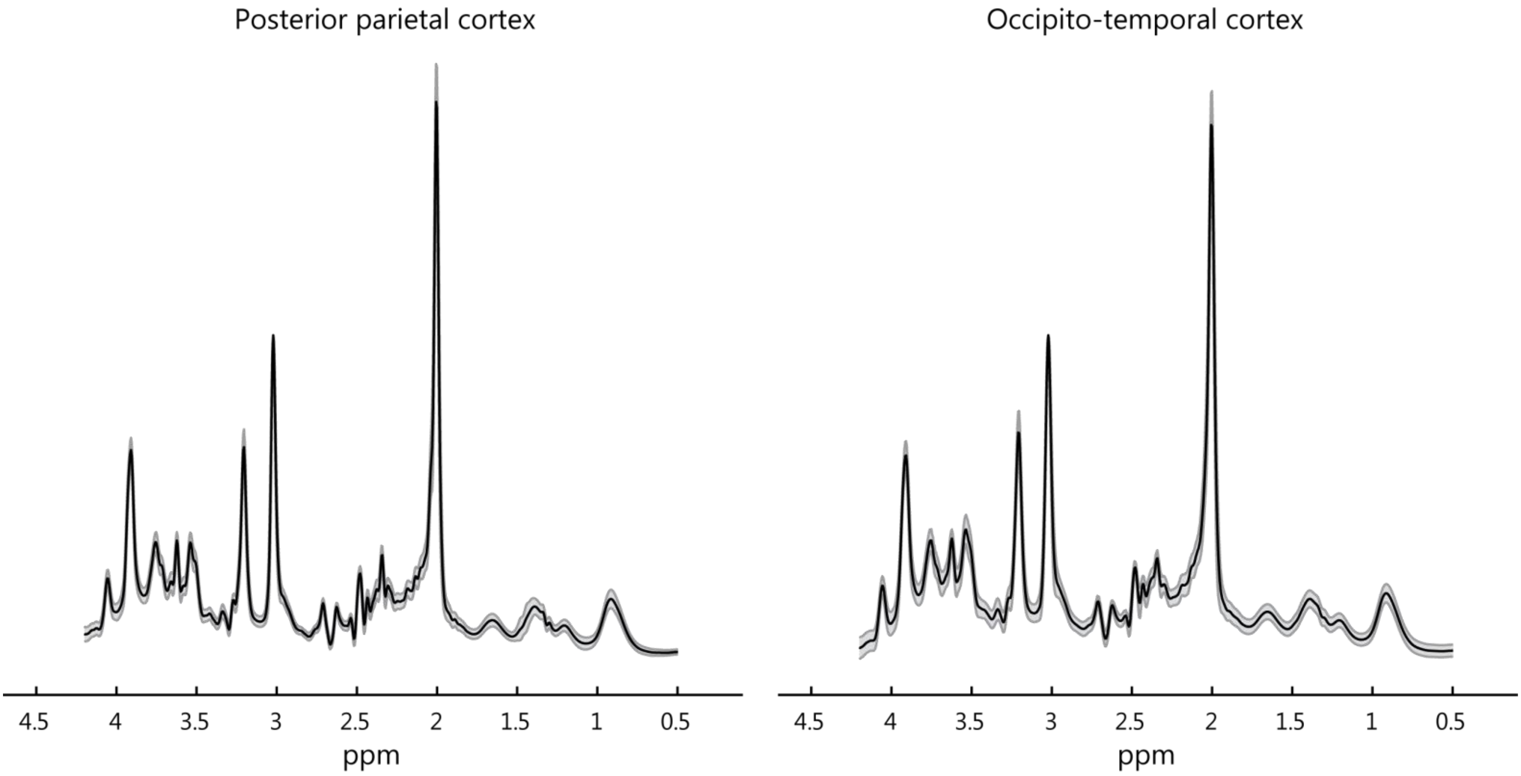

Supplementary Figure 1: Average MRS Spectrum across participants

Mean (solid black line) ± standard deviation (shade) of summed spectra (TR = 5.010 s, TE = 36 ms, number of transients = 64) from the OCT and PPC voxel across participants for the baseline measurement. The spectra are normalized to the Creatine signal from the same voxel.

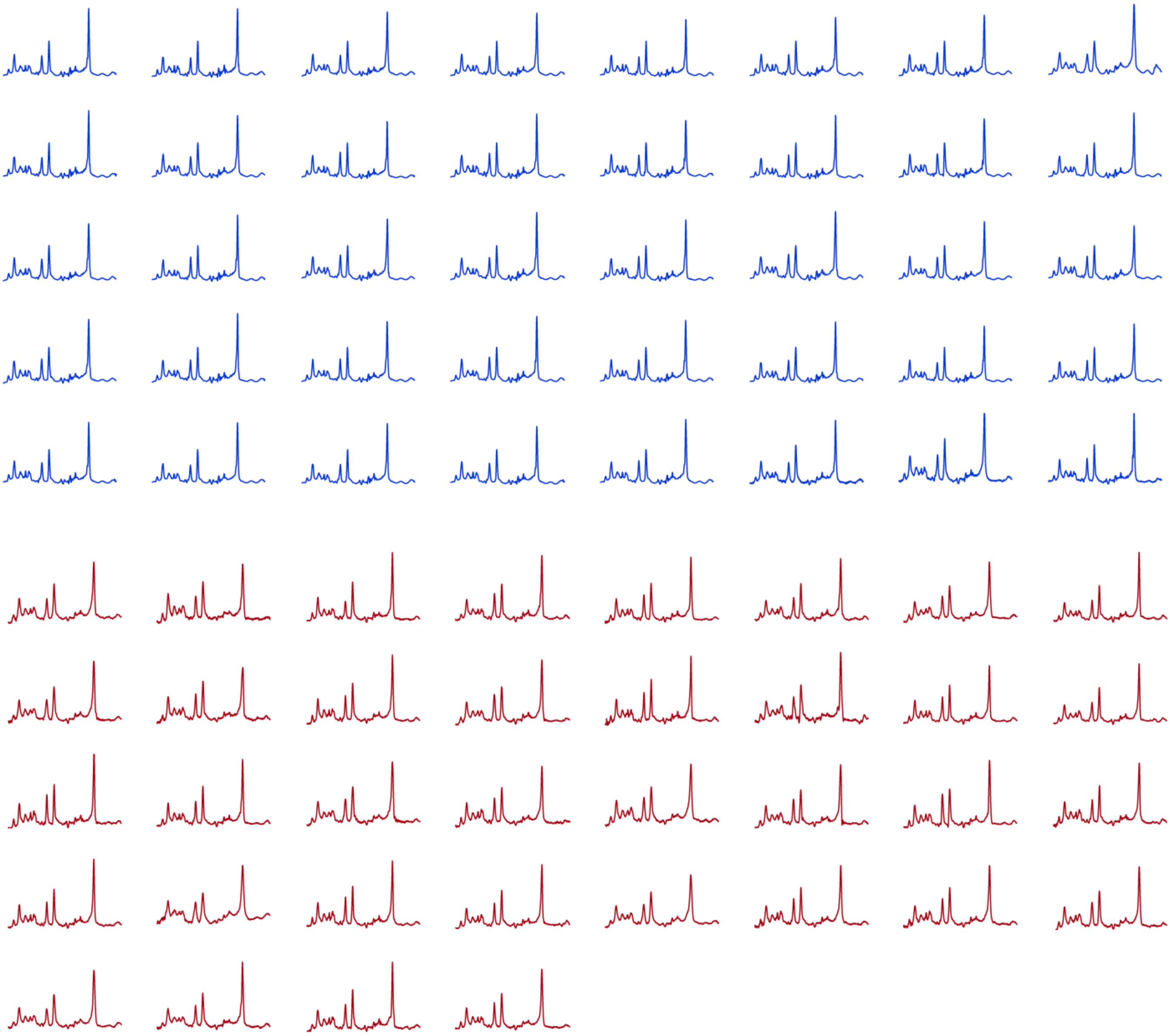

Supplementary Figure 2: Individual participant MRS spectra

Individual participant fitted MRS spectra from the OCT (in red) and PPC (in blue) voxel for the baseline measurement.

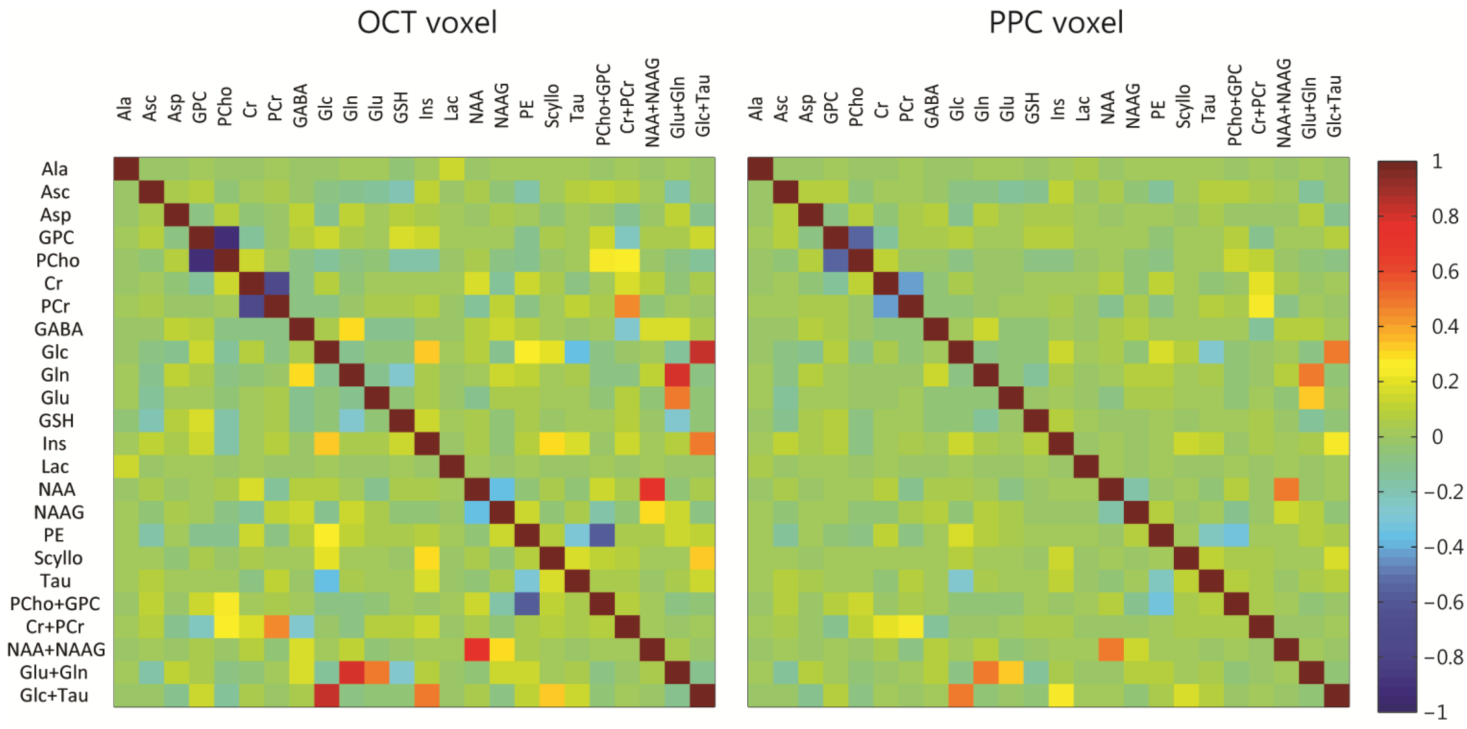

Supplementary Figure 3: Correlation coefficients across metabolites

To quantify the separation between metabolites, we evaluated the correlation coefficients between metabolite fits, as calculated by LC Model during fitting. The mean correlation coefficient between GABA and all other metabolites was always greater than -0.30 [3,34,35] for both voxels, suggesting that GABA was separable from other metabolite concentrations in our MRS measurements.

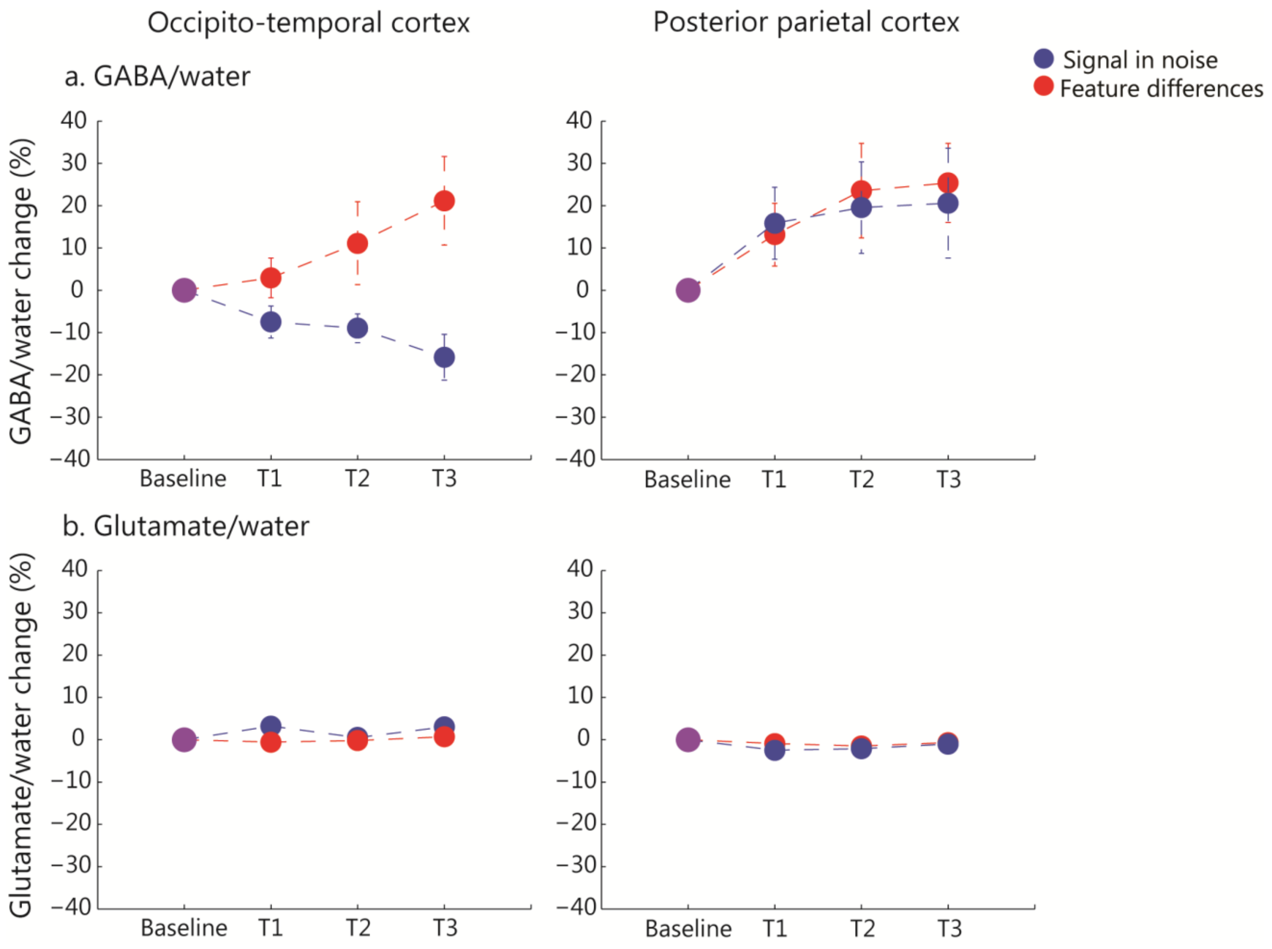

Supplementary Figure 4: Measurements of GABA/Water and Glutamate/Water during training

a. MRS-measured GABA over time is shown from two voxels (occipito-temporal, posterior parietal cortex) per task (Signal in noise, Feature differences). For each MRS-voxel, we calculated % GABA change: we normalized GABA/water per training block (T1, T2, T3) to GABA/water recorded during the baseline block; that is, we computed GABA/water change subtracting GABA/water measurements in each of the three training blocks from the baseline block and then divided by GABA/water in the baseline block. **b.** MRS-measured Glutamate over time is shown from two voxels (occipito-temporal, posterior parietal cortex) per task (Signal-in-noise, Feature differences). For each MRS-voxel, we calculated % Glutamate change: we normalized Glutamate/water per training block (T1, T2, T3) to Glutamate/water recorded during the baseline block; that is, we computed Glutamate/water change subtracting Glutamate/water measurements in each of the three training blocks from the baseline block and then divided by Glutamate/water in the baseline block. Error bars indicate standard error of the mean across participants. Error bars are not visible for small changes in metabolite concentrations.

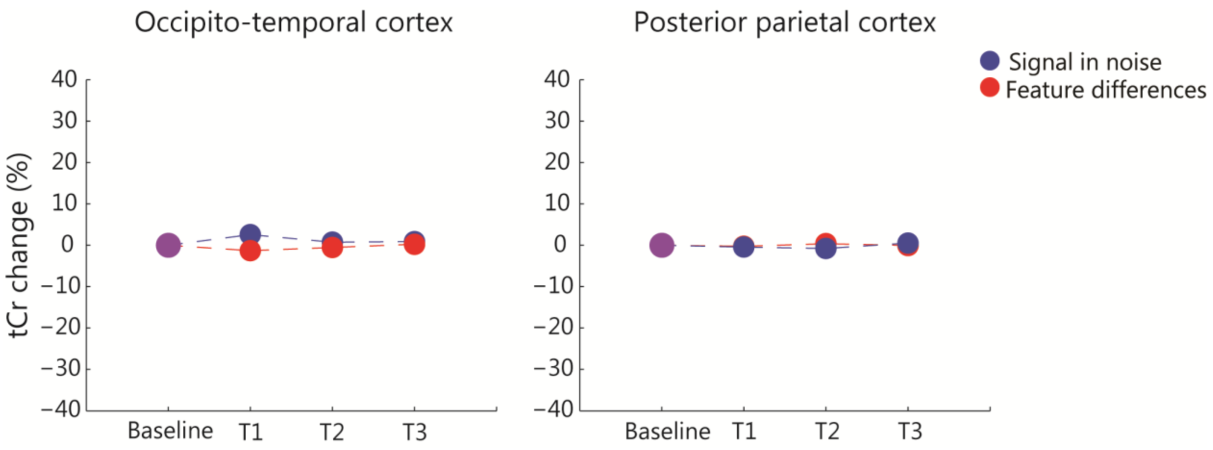

Supplementary Figure 5: Measurements of tCr during training

MRS-measured tCr over time is shown from two voxels (occipito-temporal, posterior parietal cortex) per task (Signal in noise, Feature differences). For each MRS-voxel, we calculated % tCr change: we normalized tCr per training block (T1, T2, T3) to tCr recorded during the baseline block; that is, we computed tCr change subtracting tCr measurements in each of the three training blocks from the baseline block and then divided by tCr in the baseline block. Error bars indicate standard error of the mean across participants. Error bars are not visible for small changes in metabolite concentrations.

Supplementary references

1. Öz, G. & Tkáč, I. Short-echo, single-shot, full-intensity proton magnetic resonance spectroscopy for neurochemical profiling at 4 T: Validation in the cerebellum and brainstem. *Magn. Reson. Med.* **65,** 901–910 (2011).

2. Lemke, C. *et al.* Two-voxel spectroscopy with dynamic B 0 shimming and flip angle adjustment at 7 T in the human motor cortex. *NMR Biomed* **28,** 852–860 (2015).

3. Lunghi, C., Emir, U. E., Morrone, M. C. & Bridge, H. Short-Term Monocular Deprivation Alters GABA in the Adult Human Visual Cortex. *Curr. Biol.* **25,** 1496–1501 (2015).

4. Terpstra, M. *et al.* Test-retest reproducibility of neurochemical profiles with short-echo, single-voxel MR spectroscopy at 3T and 7T. *Magn. Reson. Med.* **76,** 1083–1091 (2016).

5. van de Bank, B. L. *et al.* Multi-center reproducibility of neurochemical profiles in the human brain at 7T. *NMR Biomed.* **28,** 306–316 (2015).

6. Tkáč, I., Starčuk, Z., Choi, I. Y. & Gruetter, R. In vivo 1H NMR spectroscopy of rat brain at 1 ms echo time. *Magn. Reson. Med.* **41,** 649–656 (1999).

7. Lemke, C. *et al.* Two-voxel spectroscopy with dynamic B 0 shimming and flip angle adjustment at 7 T in the human motor cortex. *NMR Biomed* **28,** 852–860 (2015).

8. Gruetter, R. & Tkáč, I. Field mapping without reference scan using asymmetric echo-planar techniques. *Magn. Reson. Med.* **43,** 319–323 (2000).

9. Cabanes, E., Confort-Gouny, S., Le Fur, Y., Simond, G. & Cozzone, P. J. Optimization of Residual Water Signal Removal by HLSVD on Simulated Short Echo Time Proton MR Spectra of the Human Brain. *J. Magn. Reson.* **150,** 116–125 (2001).

10. Provencher, S. W. Automatic quantitation of localized in vivo 1H spectra with LCModel. *NMR Biomed.* **14,** 260–264 (2001).

11. Govindaraju, V., Young, K. & Maudsley, A. A. Proton NMR chemical shifts and coupling constants for brain metabolites. *NMR Biomed* **13,** 129–153 (2000).

12. Bednařík, P. *et al.* Neurochemical and BOLD responses during neuronal activation measured in the human visual cortex at 7 Tesla. *J. Cereb. Blood Flow Metab.* **35,** 601–610 (2015).

13. Stagg, C. & Rothman, D. *Magnetic resonance spectroscopy : tools for neuroscience research and emerging clinical applications*. (Academic Press, 2013).

14. Bogner, W. *et al.* In vivo quantification of intracerebral GABA by single-voxel 1H-MRS-How reproducible are the results? *Eur. J. Radiol.* **73,** 526–531 (2010).

15. Donahue, M. J., Near, J., Blicher, J. U. & Jezzard, P. Baseline GABA concentration and fMRI response. *Neuroimage* **53,** 392–398 (2010).

16. Sampaio-Baptista, C. *et al.* Changes in functional connectivity and GABA levels with long-term motor learning. *Neuroimage* **106,** 15–20 (2015).

17. Kühn, S. *et al.* Neurotransmitter changes during interference task in anterior cingulate cortex: evidence from fMRI-guided functional MRS at 3 T. *Brain Struct. Funct.* 2541–2551 (2015). doi:10.1007/s00429-015-1057-0

18. Kolasinski, J. *et al.* A Mechanistic Link from GABA to Cortical Architecture and Perception. *Curr. Biol.* (2017). doi:10.1016/j.cub.2017.04.055

19. Vergara, V. M., Mayer, A. R., Damaraju, E., Hutchison, K. & Calhoun, V. D. The effect of preprocessing pipelines in subject classification and detection of abnormal resting state functional network connectivity using group ICA. *Neuroimage* **145,** 365–376 (2017).

20. Patel, A. X. *et al.* A wavelet method for modeling and despiking motion artifacts from resting-state fMRI time series. *Neuroimage* **95,** 287–304 (2014).

21. Griffanti, L. *et al.* ICA-based artefact removal and accelerated fMRI acquisition for improved resting state network imaging. *Neuroimage* **95,** 232–247 (2014).

22. Murphy, K., Birn, R. M. & Bandettini, P. A. Resting-state fMRI confounds and cleanup. *Neuroimage* **80,** 349–359 (2013).

23. Van Dijk, K. R. A. *et al.* Intrinsic Functional Connectivity As a Tool For Human Connectomics: Theory, Properties, and Optimization. *J. Neurophysiol.* **103,** 297–321 (2010).

24. Bachtiar, V., Near, J., Johansen-Berg, H. & Stagg, C. Modulation of GABA and resting state functional connectivity by transcranial direct current stimulation. *Elife* **4,** 1–9 (2015).

25. Campbell, K. L. *et al.* Robust Resilience of the Frontotemporal Syntax System to Aging. *J. Neurosci.* **36,** 5214–5227 (2016).

26. Sherman, L. E. *et al.* Development of the Default Mode and Central Executive Networks across early adolescence: A longitudinal study. *Dev. Cogn. Neurosci.* **10,** 148–159 (2014).

27. Stagg, C. *et al.* Local GABA concentration is related to network-level resting functional connectivity. *Elife* **2014,** 1–9 (2014).

28. Zhu, X.-H. & Chen, W. Observed BOLD effects on cerebral metabolite resonances in human visual cortex during visual stimulation: A functional 1H MRS study at 4 T. *Magn. Reson. Med.* **46,** 841–847 (2001).

29. Kreis, R. The trouble with quality filtering based on relative Cramer-Rao lower bounds. *Magn. Reson. Med.* **75,** 15–18 (2016).

30. Emir, U. E. & Tuite, P. J. Elevated Pontine and Putamenal GABA Levels in Mild- Moderate Parkinson Disease Detected by 7 Tesla Proton. *PLoS One* **7,** 1–8 (2012).

31. Provencher, S. *LCModel & LCMgui User's Manual*. (2018). at <http://s-provencher.com/lcm-manual.shtml>

32. Marjańska, M. *et al.* Localized 1H NMR spectroscopy in different regions of human brain in vivo at 7 T: T2 relaxation times and concentrations of cerebral metabolites. *NMR Biomed.* **25,** 332–9 (2012).

33. Andreychenko, A., Klomp, D. W. J., de Graaf, R. A., Luijten, P. R. & Boer, V. O. In vivo GABA T 2 determination with J -refocused echo time extension at 7 T. *NMR Biomed.* **26,** 1596–1601 (2013).

34. Provencher, S. W. Estimation of metabolite concentrations from localized in vivo proton NMR spectra. *Magn. Reson. Med.* **30,** 672–679 (1993).

35. Tkác, I., Oz, G., Adriany, G., Uğurbil, K. & Gruetter, R. In vivo 1H NMR spectroscopy of the human brain at high magnetic fields: metabolite quantification at 4T vs. 7T. *Magn. Reson. Med.* **62,** 868–79 (2009).
